# Calprotectin and aMMP-8 as biomarkers in gingival crevicular fluid in geriatric inpatients

**DOI:** 10.3389/fdmed.2026.1790103

**Published:** 2026-04-10

**Authors:** Alicia Maria Meyer-Hofmann, Gabriele Röhrig, Steffen Büttner, Sonja Henny Maria Derman, Anna Greta Barbe

**Affiliations:** 1 University of Cologne, Faculty of Medicine and University Hospital Cologne, Polyclinic for Operative Dentistry and Periodontology, Cologne, Germany; 2 Hochschule Fresenius, Carl Remigius Medical School, Cologne, Germany, Germany

**Keywords:** cross-sectional studies, geriatric dentistry, leukocyte L1 antigen complex, matrix metalloproteinase 8, periodontitis

## Abstract

**Background:**

Accurate detection of periodontal inflammation and tissue degradation remains challenging in frail, hospitalized older adults with high periodontitis prevalences, where conventional diagnostics are often not feasible. Biochemical biomarkers such as Calprotectin (CP) and Active Matrix Metalloproteinase-8 (aMMP-8) in Gingival Crevicular Fluid (GCF) may offer low-barrier, bedside-compatible diagnostic alternatives for the future. While aMMP-8 is already well-studied and rather reflects collagen degradation, CP is supposed to indicate neutrophil-driven inflammation.

**Methods:**

In this cross-sectional study of 30 neurogeriatric inpatients (mean age 79 ± 6 years) with minimal systemic inflammation (CR*P* < 5 mg/dL), GCF samples were collected at bedside to assess aMMP-8 and CP levels and analyzed in addition to periodontal parameters [Probing Pocket Depth [PPD], Bleeding On Probing [BoP], Clinical Attachment Loss] and systemic status (blood values). Correlation and regression analyses were performed to explore associations between biomarkers and clinical indices. Correlation benchmarks were set as r = .10 weak, r = .30 moderate, r = .50 strong correlations.

**Results:**

CP and aMMP-8 showed a strong correlation (rs = .521), suggesting complementary diagnostic value. CP correlated moderately with BoP (rs = .365), mean PPD (rs = .455) and mean PPD at the sampling sites (rs = .478). aMMP-8 correlated moderately with BoP (rs = .329) and mean PDD (rs = .309). Both biomarkers were not associated with systemic variables. ANOVA revealed an effect of BoP on CP levels (*p* < .05). *post hoc* analysis showed higher CP higher in patients with BoP >30% compared to those with BoP >10%–30%. No group differences of aMMP-8 levels were observed across the BoP categories.

**Conclusion:**

Both biomarkers demonstrate a feasibility assessment for geriatric inpatients. In this population, CP reflects localized periodontal processes and inflammation. CP may in the future be particularly suited for inflammatory screening in care-dependent older adults. These findings contribute initial reference data and underscore the need for larger studies to validate biomarker-based diagnostics in broader geriatric populations.

## 1 Introduction

Accurate and practical detection of periodontal inflammation and connective tissue degradation remains a central challenge in geriatric dentistry, particularly within the context of outreach dental care. It is well established that the prevalence of periodontitis is notably high in elderly populations, a phenomenon partially attributable to declining oral hygiene capacity, reduced access to dental services, and the long-term retention of natural teeth due to earlier preventive dental care (1). While comprehensive periodontal diagnostics such as pocket depth measurement and clinical index scoring are standard in ambulatory dental settings, their implementation becomes highly impractical in frail, homebound, or hospitalized geriatric patients (2, 3). Limitations such as restricted duration of mouth opening capability, poor lighting conditions, and patient cooperation at the bedside significantly impede conventional clinical assessments (4).

Yet, the clinical imperative for effective periodontal diagnostics in this population continues to grow. Evidence increasingly supports a link between oral inflammation and systemic health outcomes, emphasizing the need for reliable, low-threshold diagnostic approaches especially in advanced age (5–7). Furthermore, as healthcare models evolve toward integrated, interprofessional pathways involving medicine, nursing, and dentistry, standardized diagnostic frameworks suitable for bedside application will be essential for cross-sectoral coordination and patient-centered care.

The diagnosis of periodontal and peri-implant diseases is based on clinical parameters and indices, including Probing Pocket Depth (PPD), Bleeding On Probing (BoP), and Clinical Attachment Level (CAL), complemented by radiographic assessment (8). These measures accurately capture the current extent and severity of disease (9). However, in situations where conventional assesments cannot be performed, additional approaches are needed. Consequently, biomarker analysis in Gingival Crevicular Fluid (GCF) and saliva may represent a valuable adjunct, offering a non or minimal-invasive approach to detect ongoing inflammatory processes, to monitor inflammation and to potentially predict disease progression (10, 11). Salivary or GCF sampling could provide a rapid, non-invasive indication of oral inflammatory status (12).

Two biomarkers have emerged as promising in recent years: Active Matrix Metalloproteinase-8 (aMMP-8) has gained much scientific attention and, more recently, Calprotectin (CP). aMMP-8 is a collagen-degrading enzyme secreted primarily by neutrophilic granulocytes and serves as a robust indicator of active extracellular matrix degradation (13, 14). Its elevated concentrations in GCF and saliva strongly correlate with ongoing periodontal tissue destruction, thereby enabling early detection and longitudinal monitoring via chairside assays (15, 16). CP, a heterodimer of the S100A8 and S100A9 proteins, is likewise released by mainly neutrophils during contact with inflamed endothelium and plays an important role in inflammatory process by promoting leukocyte adhesion, activating immune cells, and limiting bacterial growth via zinc sequestration (17–19). Within the pathogenic cascade of periodontal disease, CP is released early as a marker of acute inflammatory activity, whereas aMMP-8 appears later as an effector molecule of collagen breakdown, thus reflecting subsequent structural tissue destruction (20, 21).

While both biomarkers hold diagnostic promise, it is important to note that neither aMMP-8 nor CP is currently integrated into standard periodontal diagnostic protocols for all age groups. However, point-of-care test of aMMP-8 assays have been introduced into clinical practice and are commercially available as adjunctive tools for detection of periodontal destruction, yet their uptake remains limited (22, 23). CP, by contrast, has achieved greater clinical traction in other medical disciplines: fecal CP is routinely employed in gastroenterology to monitor inflammatory bowel diseases such as Crohn's disease and ulcerative colitis, and serum CP is increasingly used in rheumatology to assess disease activity in rheumatoid arthritis (24).

Against this background, the present pilot analysis offers a first exploratory investigation into the relationship between aMMP-8 and CP and the association with systemic health in the context of hospitalized geriatric patients with care needs. We hypothesize that these biomarkers may reflect complementary dimensions of periodontal disease activity and exhibit correlative behavior. Our analysis also seeks to firstly determine whether these markers maintain diagnostic validity in a hospital-based elderly cohort and how the obtained values compare to those reported in other populations. All study participants presented with minimal systemic inflammation, as indicated by low C-reactive protein (CRP) levels, enabling interpretation of aMMP-8 and CP concentrations as representative of destructive periodontal processes. These findings of this cross-sectional study may help lay the groundwork for biomarker-based diagnostics in multidisciplinary geriatric care frameworks, especially where conventional clinical diagnostics are constrained.

## 2 Materials and methods

### 2.1 Ethical approval

This secondary analysis was conducted within the framework of a previously approved observational study (Ethics Committee of the Medical Faculty, University of Cologne; Approval No. 20-1721). The study was prospectively registered in the German Clinical Trials Register (DRKS00024446, registration date: 11 June 2021). All study procedures adhered to the Declaration of Helsinki, and written informed consent was obtained from all participants prior to any study-related assessments. This study was reported in accordance with the STROBE guidelines for observational studies (25). Additional analyses based on the existing dataset were covered by the original ethical approval. The primary study evaluated the oral health of neurogeriatric inpatients without systemic inflammation and the association with hematological parameters representing systemic health. Further details regarding the primary study protocol can be found in the associated publication (26).

### 2.2 Study population and setting

This cross-sectional study included a subset of hospitalized individuals aged over 70 years who were undergoing early neurological rehabilitation at St. Marien-Hospital in Cologne, Germany, between September 2021 and October 2022. Participants were selected for inclusion based on the absence of acute or chronic systemic inflammation, verified through low CRP levels (≤5 mg/dL). To minimize potential confounding, an interdisciplinary expert panel—comprising specialists in dentistry, neurology, and geriatrics—evaluated patient records to confirm the lack of alternative sources of low-grade systemic inflammation beyond the oral cavity. Exclusion criteria encompassed active malignancies requiring treatment, current or recent infections, use of antibiotics at the time of assessment, chronic inflammatory conditions (e.g., rheumatoid arthritis, inflammatory bowel disease), recent ischemic stroke treated with thrombolysis, impaired renal function (eGFR <30 mL/min or dialysis), and recent cardiovascular interventions.

### 2.3 Data collection and bedside oral examination

All clinical oral examinations were performed by the first author (A.M.MH) at the patient's bedside under conditions dictated by hospital hygiene and pandemic-related access restrictions. Due to infection control measures during the COVID-19 pandemic, no dental assistant was permitted during examinations; findings were dictated in real-time and transcribed retrospectively. Examination conditions included the use of magnifying loupes with integrated lighting to optimize visualization in suboptimal clinical environments.

Oral assessments included the number of remaining natural teeth, prosthetic status, and plaque levels according to the Silness and Löe Plaque Index (27) (PI). Periodontal probing was limited to the mesiobuccal and distobuccal sites of each tooth to determine PPD, gingival recession, and CAL. BoP was recorded on mesiobuccal and distobuccal sites as a measure of current inflammatory activity. Additional clinical signs such as furcation involvement (28) and tooth mobility (29) were noted, and periodontal diagnoses were classified according to both the AAP/EFP 2018 (30) classification and the earlier CDC/AAP system (31). The staging (EFP/AAP) and clinical case definition (AAP/CDC) are available in the Supplementary Material.

### 2.4 Biomarker sampling at the bedside

For this secondary analysis, additional GCF samples were obtained to quantify two key biochemical markers: aMMP-8 and CP. Sampling was performed at the patient's bedside immediately following periodontal probing. Sterile GCF/PISF Collection Stripes (Dentognostics GmbH, Jena, Germany) were used for aMMP-8 collection, and paper points ISO 50 (Reciproc, VDW, Munich, Germany) were used for CP collection in GCF. The collection strips and paper points were inserted into the gingival sulcus of the deepest pocket per quadrant for 30 seconds, one after another. The pockets were selected based on the greatest probing depth and absence of visible bleeding. GCF samples were collected for aMMP-8 and CP and stored separately in Eppendorf tubes. Paper strips contaminated with blood or pus were discarded. The procedure was conducted under aseptic conditions without local anesthesia, within the constraints of a mobile care setting. For each aMMP8 and CP analysis, samples from the four sampling sites of each participant were pooled. All samples were handled and processed according to the manufacturer's guidelines. A commercially available sandwich ELISA kit (Pool DPS Test, Dentognostics GmbH, Jena, Germany) was used for the quantitative analysis of aMMP-8. An immunodiagnostic kit (IDK Calprotectin ELISA, Immundiagnostik AG, Bensheim, Germany), which was originally designed for the *in vitro* determination of calprotectin (MRP8/14, S100A8/A9) in stool samples, was used for the CP analysis.

### 2.5 Systemic and functional parameters

Demographic and clinical data, including age, sex, comorbidities, and current medications, were extracted from patient records. Functional status at admission was assessed using the Barthel Index (32), with particular focus on the “grooming” subscore as an indirect indicator of oral self-care capacity. Hematological parameters such as CRP, white blood cell count, hemoglobin, ferritin, total protein, albumin, vitamin B12, and folic acid were retrieved from routine hospital laboratory reports to evaluate systemic inflammation, anemia, and nutritional status. Those parameters have been published previously (26).

### 2.6 BoP grouping

The extent of inflammation was determined based on the percentage of BoP. The grouping was made according to the threshold values proposed by Chapple et al. (33) in <10%, 10%–30% and ≥ 30% BoP. This classification was used to categorize the extent of inflammation, regardless of the presence of periodontitis.

### 2.7 Statistical analysis

The sample size is based on the research question of the primary analysis and is explained in the associated publication (26). Also clinical characteristics of the study population, including periodontal indices, have been reported in detail in this primary publication. For the present secondary analysis, all continuous variables were re-evaluated for normality using the Shapiro–Wilk test. Based on the distribution of the data, either Pearson's product-moment correlation or Spearman's rank correlation coefficients were computed to assess the strength and direction of associations between biomarker concentrations (aMMP-8, CP) and clinical periodontal parameters, including PPD, CAL and BoP. The classification of correlation strengths was based on the benchmarks according to Cohen's guidelines: r = .10 (weak), r = .30 (moderate), and r = .50 (strong). A one-way ANOVA or Welch's ANOVA was used to compare GCF aMMP-8 and CP levels across the three BoP groups, with the specific method chosen depending on the assumption of homogeneity of variances. *post hoc* tests and *η*^2^ were used for further group differentiation. In addition, exploratory multiple linear regression models were constructed to examine the extent to which periodontal parameters predicted the concentrations of aMMP-8 and CP, respectively. Multicollinearity was assessed using variance inflation factors, and model fit was evaluated using adjusted R^2^ values. Statistical significance was defined as *p* < 0.05. All statistical analyses were performed using IBM SPSS Statistics for Windows, Version 28.0 (IBM Corp., Armonk, NY, USA).

## 3 Results

### 3.1 Patient characteristics

A total of 30 patients (15 male and 15 female, average age 79 ± 6 years) with an average of 7 ± 3 comorbidities and existing polypharmacy were analyzed. Neurogeriatric inpatients showed a low mean Barthel Index of 31 ± 18, indicating high dependency in activities of daily living. Laboratory analyses revealed frequent abnormalities in blood values, including reduced levels of red blood cells, haemoglobin, haematocrit and total protein, as well as elevated levels of CRP and ferritin. 73% of patients met the World Health Organization's criteria for anemia (34). Mean PPD at the sampling sites was 4.4 ± 1.2 mm (range 3.0–7.0 mm). The concentration of aMMP-8 in GCF ranged from 0.0 to 80.0 ng/mL, with a mean value of 15.1 ± 15.7 ng/mL. CP levels in sulcus fluid varied between 432 and 6,828 ng/mL, with a mean of 2,443.3 ± 1,580.5 ng/mL (Table 1).

**Table 1:** Clinical patient characteristics and periodontal parameters.

Characteristics of *N* = 30 geriatric inpatients	Mean ± SD	Range
Age (years)	79 ± 6	70–90
BI (at admission)	31 ± 18	10–75
BoP (%)	26 ± 17	7–69
Mean PPD (mm)	3.1 ± 0.7	2.2–4.9
Mean PPD sampling site (mm)	4.4 ± 1.2	3.0–7.0
Max PPD sampling site (mm)	5.6 ± 2.4	3–14
GCF aMMP8 (ng/mL)	15.1 ± 15.7	0.0–80.0
GCF CP (ng/mL)	2,443.3 ± 1,580.5	432–6,828

aMMP-8, active matrix metalloproteinase-8; BI, Barthel index; BoP, bleeding on probing; CP, calprotectin; GCF, gingival crevicular fluid; PPD, probing pocket depth; SD, standard deviation.

Correlation analysis revealed a strong association between aMMP-8 and CP levels. CP showed a moderate positive correlation with BoP, mean PPD, max PPD, mean PDD at the assessed sampling sites and max PPD at the assessed sampling sites. aMMP-8 levels showed a moderate association with BoP and mean PPD. Neither biomarker showed moderate or strong associations with CAL, PI or hematological parameters representing systemic inflammation (Table 2).

**Table 2:** Spearman's Rho correlations between dental, periodontal or functional parameters and GCF concentrations of aMMP-8 and calprotectin for *N* = 30 geriatric inpatients.

Parameter	GCF CP (ng/mL)	GCF aMMP-8 (ng/mL)
GCF CP (ng/mL)	1	.521**
GCF aMMP-8 (ng/mL)	.521**	1
CAL (mm)	.112	.088
BoP (%)	.365*	.**329**
Mean PPD (mm)	.455*	.**309**
Max PPD (mm)	.**353**	.198
Mean PPD sampling site (mm)	.478**	.244
Max PPD sampling site (mm)	.**353**	.198
Plaque index	-.082	-.050
CRP (mg/dL)	-.012	-.250
WBC (1/nL)	.136	.122

aMMP-8, active matrix metalloproteinase-8; BoP, bleeding on probing; CAL, clinical attachment level; CP, calprotectin; CRP, C-Reactive Protein; GCF, gingival crevicular fluid; PPD, probing pocket depth; SD, standard deviation, WBC, white blood cell count. Bold values indicate moderate or strong correlations according to Cohens guidelines.

* *p*-value < .05.

** *p*-value < .01.

### 3.2 BoP grouping and comparison

The predefined BoP categories reflected progressively higher mean BoP values, consistent with the grouping criteria. Mean BoP in the BoP >30% was 46.6 ± 13.5% (Table 3).

**Table 3:** Comparison of BoP, GCF calprotectin, and GCF aMMP-8 levels among groups stratified by BoP.

Parameter	BoP < 10% *n* = 5	BoP 10%–30% *n* = 15	BoP > 30% *n* = 10	*p*- value	Test
BoP (%), mean ± SD	8.7 ± 1.4.	19.0 ± 5.4	46.6 ± 13.5	**<.001**	Welch-ANOVA
GCF CP (ng/mL), mean ± SD	1,701.4 ± 1,181.2	1,988.4 ± 1,082.5	3,496.5 ± 1,929.6	**.027**	ANOVA
GCF aMMP-8 (ng/mL), mean ± SD	9.1 ± 6.5	10.7 ± 6.4	24.8 ± 23.6	.052	Welch-ANOVA

aMMP-8, active matrix metalloproteinase 8; BoP, bleeding on probing; CP, calprotectin; GCF, gingival crevicular fluid; SD, standard deviation. Bold: statistically significant. Significance level *p* < .05.

#### 3.2.1 Calprotectin

The analyses of variance (ANOVA) revealed a significant effect of BoP on the CP levels in GCF [F(2, 27) = 4.123, *p* < .05]*.* This indicates that there are significant differences in the mean values of GCF CP levels across the three BoP groups (Table 3). The *post hoc* analysis revealed significant differences (*p* < .05) between the GCF CP levels of the groups with BoP >10%–30% and BoP >30% [1,508.1, 95% confidence interval (CI) 56.91, 2,959.3]. The corresponding *η*2 showed a large effect of .234, according to Cohen's guidelines.

#### 3.2.2 aMMP-8

GCF aMMP-8 levels were normally distributed for BoP ≤10% and BoP >10%–30%, but not for BoP >30%, as assessed by the Shapiro–Wilk test (*α* = .05). Homogeneity of variances was asserted using Levene's Test which showed that no equal variances could be assumed (*p* = .006). The Welch-ANOVA revealed a non-significant effect of BoP on the aMMP-8 levels in GCF (*p* > .05) (Table 3).

### 3.3 Predictors of CP and aMMP-8

The linear regression model indicated a statistically significant and moderate proportion of variance of CP in GCF [R^2^ = .32, F(3,26) = 4.04, *p* < .05, adjusted R^2^ = .24]. Among the predictors, only mean PPD sampling sites was a significant positive predictor of CP in GCF (*p* = .028). BoP (*p* = .098) and plaque index (PI) (*p* = .269) showed positive, but non-significant associations.

The linear regression model including BoP, mean PPD sampling sites, and PI did not significantly predict aMMP-8 levels [R^2^ = .20, F(3,26) = 2.14, *p* >.05, adjusted R^2^ = .11]. None of the predictors (mean PPD sampling sites, BoP, PI) reached statistical significance (*p* < .05).

## 4 Discussion

This pilot study demonstrates that aMMP-8 and CP in GCF reflects aspects of periodontal disease activity in older hospitalized neurogeriatric patients, with CP emerging as a more inflammation-sensitive marker compared to aMMP-8 in GCF in this population. Both biomarkers showing independence from systemic confounders represented by inflammatory blood markers in a systemically well characterized population with low grade systemic inflammation due to exclusion criteria.

Previous reports have highlighted aMMP-8 in oral fluids as a promising biomarker for distinguishing between active and inactive periodontal disease, thus supporting its potential application in periodontal diagnostics (14, 16, 22). This cohort showed an average of 15.1 ng/mL of aMMP8 in GCF, as did a study of patients with periodontal disease and healthy individuals, in which the patients with periodontal disease had an average of 14.3 ng/mL of aMMP8 in contrast to the healthy ones with 1 ng/mL (35). Our findings suggest that CP in GCF could provide a more reliable reflection of periodontal status especially when assessing and monitoring the degree of inflammation. These results suggest that CP may be a more sensitive marker of local periodontal inflammation in this cohort.

Periodontal inflammation is closely associated with increased infiltration of neutrophil granulocytes, which are the main source of CP in the GCF (18). The absence of a significant difference in CP levels between groups with BoP of less than 10% and between 10% and 30% may indicate that mild increases in periodontal inflammation do not yet lead to substantial changes in neutrophil-derived inflammatory activity. By contrast, CP levels were higher in the group with BoP >30%, suggesting that higher levels correspond to stronger effects whereby CP in GCF only becomes markedly elevated once periodontal inflammation exceeds a certain level. However, this pattern may be influenced by group size and variance, which limits the statistical power to detect smaller differences in the lower BoP categories.

An important advantage of the present approach is the quantitative analysis of exact aMMP-8 and CP concentrations in GCF. Most commercially available point-of-care tests for aMMP-8 only provide dichotomous outcomes (negative vs. positive) in relation to predefined thresholds, without determining precise concentration levels (36–39). While these screening tests are useful for chairside diagnostics, they do not permit a detailed assessment of how biomarkers change across the different stages of periodontal inflammation (22). In contrast, the present analysis provides exact concentration values, thereby enabling the identification of clinically meaningful ranges and thresholds. This adds novel insights into the diagnostic value of CP as a potential biomarker of periodontal and systemic inflammation.

Unlike aMMP-8, for which reference values and clinical cut-offs have been defined in various populations and are available in multiple diagnostic devices, CP lacks established normative ranges for oral applications. There are a few studies examining CP in GCF in several study populations with generalized periodontitis but also periodontal healthy (40, 41). These studies support the use of CP as a promising biomarker for diagnosing and monitoring periodontal disease. However, the data from these studies is insufficient to determine cut-off values for oral inflammation, particularly among older people. This group remains underrepresented in research, especially in basic research and translational studies. Existing reference values for CP are predominantly based on fecal or serum measurements in the context of systemic inflammatory disorders and may not be transferrable to the periodontal microenvironment. The present study contributes preliminary data toward defining population-specific reference levels for oral CP in a hospitalized geriatric cohort, under conditions of minimal systemic inflammation. These data may serve as a foundation for future research aimed at establishing diagnostic thresholds tailored to older persons. Ultimately, reliable reference values—stratified by age, care dependency, and comorbidity profiles—will be essential to enable valid interpretation of CP concentrations in periodontal diagnostics and to support its potential integration into interdisciplinary geriatric care models.

As individuals with elevated systemic CRP levels were excluded, the effect of systemic inflammation on biomarker levels was intentionally minimized, ensuring that periodontal inflammation remained the primary source of inflammatory markers in the sulcus fluid. Accordingly, no clear influence of oral CP or aMMP-8 on systemic inflammatory parameters, or vice versa, was observed. Future studies should therefore include participants with systemic inflammation to better explore these interactions.

Based on the information available in the manufacturer's diagnostic documentation, only qualitative inferences regarding diagnostic performance can be drawn, as the complete contingency tables required for calculating sensitivity and specificity are not reported. Nevertheless, the validation data provided by the test supplier suggest that calprotectin may outperform aMMP-8 in distinguishing periodontal health from disease. In the healthy control group, aMMP-8 showed elevated values in 3 of 8 individuals, indicating potential false-positive results and thus reduced specificity. In contrast, calprotectin did not demonstrate comparable elevations in this group. Furthermore, in patients with periodontitis (pocket depth >3 mm), all individuals had calprotectin concentrations above the diagnostic threshold, whereas aMMP-8 measurements were below the detection limit in 3 of 13 cases, representing potential false-negative results. These observations are therefore consistent with comparatively higher diagnostic sensitivity and specificity for calprotectin relative to aMMP-8 (41–45). However, these conclusions remain qualitative and should be interpreted with caution, as they are derived from manufacturer-reported validation data with limited sample size and without full diagnostic classification metrics. The manufacturer documentation refers to several studies investigating calprotectin in gingival crevicular fluid and its association with periodontal disease activity.

This study has several limitations, most of which are related to the fact that it is a secondary outcome analysis. The cross-sectional design and the relatively small sample size do not allow causal inferences to be drawn between biomarker levels and disease progression. The study population consisted of a specific cohort of hospitalized elderly patients with multimorbidity, which limits the generalizability of the results to a broader elderly population. The absence of a healthy control group and the high prevalence of periodontitis may have obscured stage-specific differences further. GCF samples were pooled from four sites, which reduces site-specific resolution and may dilute localized inflammatory activity. In addition, relying on only four sampling sites provides limited representation of the overall periodontal condition. The present study constitutes a secondary analysis of a previously published observational cohort. The sample size was based on the primary study question and was originally calculated to detect large effects (*β* or r ≥ 0.5) with 80% power at a two-sided *α*-level of 0.05. Therefore, the current biomarker analyses were not specifically powered *a priori* and should be considered exploratory. While the available sample size was likely sufficient to detect large associations, it may have been insufficient for small to moderate effects; consequently, non-significant findings should be interpreted with caution.

While aMMP-8 in oral fluids may be suitable for identifying periodontitis, the inflammatory burden and its systemic implications appear to be of greater clinical relevance in a population with a high prevalence of periodontal disease. Our findings provide initial evidence that an assessment of CP in GCF may suit as a screening tool for hospitalized elderly patients, as it provides a more direct reflection of local inflammatory status. However, further studies are needed to confirm these results, also among persons with systemic inflammation, establish clinically meaningful cut-off values and elucidate the links between oral and systemic health in elderly populations.

## 5 Conclusion

These findings provide the first evidence that CP in GCF could serve as a biomarker for periodontal inflammation in hospitalized elderly patients during bedside assessment. Further studies involving larger and more diverse populations are needed to confirm the diagnostic usefulness and practicability of these biomarker tests at the bedside, and to examine their performance in groups with concurrent systemic inflammatory conditions that could have a confounding effect.

## Data Availability

The raw data supporting the conclusions of this article will be made available by the authors upon request, without undue reservation.
