# Supplementary material for: Calprotectin and aMMP-8 as biomarkers in gingival crevicular fluid in geriatric inpatients
Source: Front Dent Med. 2026 Apr 10;7:1790103. doi: 10.3389/fdmed.2026.1790103 (PMC13106960; doi:10.3389/fdmed.2026.1790103)
Supplement: Supplementary file 1 [file Table1.docx]

Supplementary Material

Table S1: Periodontal diagnosis

| **Periodontal staging (AAP/EFP)** | **n (%)** |
| --- | --- |
| Stage I | 4 (13) |
| Stage II | 8 (27) |
| Stage III | 7 (23) |
| Stage IV | 5 (17) |
| No periodontitis | 6 (20) |
| **Periodontal case definition (CDC/AAP)** | **n (%)** |
| No periodontitis | 5 (17) |
| Mild periodontitis | 10 (33) |
| Moderate periodontitis | 7 (23) |
| Severe periodontitis | 8 (27) |

Abbreviations: AAP, American Academy of Periodontology, CDC, Centers for Disease Control and Prevention, EFP, European Federation of Periodontology
